# Stability and Rheological Behavior of Mayonnaise-like Emulsion Co-Emulsified by Konjac Glucomannan and Whey Protein

**DOI:** 10.3390/foods12152907

**Published:** 2023-07-31

**Authors:** Yaqiong Pei, Yanqiu Zhang, Hui Ding, Bin Li, Jun Yang

**Affiliations:** 1 College of Food Science and Technology, Wuhan Business University, Wuhan 430056, China; peiyaqiong@wbu.edu.cn (Y.P.); 20150481@wbu.edu.cn (H.D.); 2 College of Food Science and Technology, Huazhong Agricultural University, Wuhan 430070, China

**Keywords:** konjac glucomannan, whey protein, mayonnaise-like emulsion, stackability, rheology

## Abstract

The aim of this work was to study the physical stability and rheological properties of an oil-in-water emulsion stabilized by a konjac glucomannan–whey protein (KGM-WP) mixture at a konjac glucomannan concentration of 0.1–0.5% (*w*/*w*) and a whey protein concentration of 1.0–3.0% (*w*/*w*). The droplet size, microstructure, stackability, flow behavior, and viscoelastic properties were measured. The experimental results showed that with an increase in KGM and WP concentrations, the droplet size (D_4,3_) of the emulsion gradually decreased to 12.9 μm, and the macroscopic performance of the emulsion was a gel-like structure that can be inverted and resist flow and can also be extruded and stacked. The static shear viscosity and viscoelasticity generally increased with the increase of konjac glucomannan and whey protein concentration. Emulsions were pseudo-plastic fluids with shear thinning behavior (flow behavior index: 0.15 ≤ n ≤ 0.49) and exhibited viscoelastic behavior with a storage modulus (G′) greater than their loss modulus (G″), indicating that the samples all had gel-like behavior (0.10 < n′ < 0.22). Moreover, storage modulus and loss modulus of all samples increased with increasing KGM and WP concentrations. When the concentration of konjac glucomannan was 0.3% *w*/*w*, the emulsion had similar rheological behavior to commercial mayonnaise. These results suggested that the KGM-WP mixture can be used as an effective substitute for egg yolk to make a cholesterol-free mayonnaise-like emulsion. The knowledge obtained here had important implications for the application of protein–polysaccharide mixtures as emulsifiers/stabilizers to make mayonnaise-like emulsions in sauce and condiments.

## 1. Introduction

Mayonnaise is an oil-in-water emulsion with gel-like properties that can be manufactured by emulsifying egg yolk, oil, vinegar, and other spices [1]. Because of its excellent taste and flavor, mayonnaise has become an important food sauce. With the convenience food and prepared dish industries growing rapidly, the development and application of mayonnaise have great prospects. However, traditional mayonnaise is high in cholesterol (prepared using egg yolk) and fat (containing above 65% oil), which can increase the risk of various chronic diseases such as obesity, cardiovascular disease, and diabetes [2]. In addition, the use of egg yolk used as an emulsifier poses a risk of *Salmonella* contamination [3,4]. For these reasons, traditional mayonnaise has been unable to meet consumer demand for healthy food. Therefore, recent research has focused on optimization the of mayonnaise formulas to enhance its nutritional function and health safety.

The improvement of mayonnaise formula was mainly focused on two effective strategies. The first strategy involved partially or completely replacing the egg yolk with alternative emulsifiers to fabricate low-cholesterol or cholesterol-free mayonnaise, which addressed the issue of high cholesterol content in traditional mayonnaise. These alternative emulsifiers included small-molecule surfactants [5], animal/plant proteins [6,7], protein/polysaccharides [8], or protein/polyphenol composite particle complexes [9]. However, replacing egg yolk completely or partially could negatively affect some sensory properties, such as spreadability, mouth hardness, mouth coating, and mayonnaise surface gloss, and some physical stability characteristics, such as droplet size, microstructure, rheology features, and shelf life [10,11]. For example, the critical strain as an index of mayonnaise extensibility decreased with an increase in egg yolk content above 4%. Previous research utilized starch [12], cellulose [13], and xanthan gum [14] as emulsifying stabilizers or thickening agents to overcome these negative effects to prepare stable low-cholesterol or cholesterol-free mayonnaise with a typical gel-like property. The second strategy was employed to address the issue of high fat. This was achieved by reducing the amount of oil and using food biomacromolecules as a fat substitute to maintain the product’s quality. The use of food biomacromolecules (such as carbohydrate-, protein-, fat-, and synthetic-based fat substitutes) helped compensate for the decrease in viscosity, taste, flavor, and appearance caused by the reduction in oil [12,15,16]. Additionally, fats rich in healthy polyunsaturated fatty acids which might protect against various chronic diseases, such as fish oil, olive oil, canola oil, and flaxseed oil [7,17,18,19], were used to partially or entirely replace saturated fats. This was done in order to increase the proportion of unsaturated fatty acids and to innovate the development of mayonnaise products with both stability and functionality.

Konjac glucomannan (KGM), a water-soluble polysaccharide extracted from the tuber of amorphophallus konjac, was composed of glucose and mannose with 5–10% acetyl substitution [20]. Due to its high viscosity, excellent gelling properties, and healthy bioactivity of dietary fiber (such as preventing diabetes, obesity, and hyperglycemia), KGM had been increasingly used as a food additive or food supplement [5,21]. Whey protein was widely exploited in the food emulsification system owing to its excellent interfacial and gel properties [22,23,24]. Moreover, whey protein could be used as a fat-replacement substance in the production of reduced-calorie mayonnaise-type products [7].

Based on the above considerations, we proposed using a konjac glucomannan–whey protein (KGM-WP) mixture and flaxseed oil rich in polyunsaturated fatty acids to replace egg yolk and common edible oil, respectively, to improve the formula of traditional mayonnaise and prepare a reduced-calorie mayonnaise-like emulsion. However, the change in formulation would inevitably lead to the changes in product stability and rheology characteristics, especially the change in the amounts of egg yolk and oil, which were two main ingredients of mayonnaise [25,26]. As a science of flow and deformation, rheology played a crucial role in characterizing the quality characteristics of food emulsion. These characteristics were dependent on changes that might occur in formulation, processing parameters, and equipment [26]. These quality characteristics (rheological properties) of food emulsion were closely related to their appearance, texture, taste, and shelf-life [27]. In our previous study, impacts of flaxseed oil concentration on the physical stability and rheological properties of mayonnaise-like emulsions were investigated [28].

The objective of the current work was to investigate the impact of KGM-WP mixture concentration on the physical stability, such as appearance, droplet size, microstructure, stacking, and extrusion formability, and rheological properties of emulsion stabilized by a KGM-WP mixture. Our findings might guide the rational application of a KGM-WP mixture as an effective replacer of egg yolk to fabricate cholesterol-free mayonnaise-like emulsion. It provided valuable reference information for the processing and production of mayonnaise-like emulsion products.

## 2. Materials and Methods

### 2.1. Materials

Food-grade flaxseed oil (FO) was purchased from Xilin Gol League Hongjingyuan Grease Co., Ltd. (Inner Mongolia, China). Powdered whey protein (WP, protein content of 94%)) was purchased from Aladdin Biochemical Technology Co., Ltd. (Shanghai, China). Powdered konjac glucomannan (KGM) was purchased from Hubei Johnson Konjac Technology Co. Ltd. (Wuhan, China). White vinegar, sugar, and salt used as condiments were all food-grade and were purchased from local supermarkets. All samples were prepared with double-distilled water (DD water).

### 2.2. Preparation of Mayonnaise-like Emulsion

Initially, a 30% *w*/*w* WP stock solution was prepared by adding WP powder into water and stirring (600 rpm) at 25 °C for 2 h, and it was then stored at 4 °C for 8 h to ensure adequate hydration of protein molecules. A 5% *w*/*w* KGM stock solution was prepared by adding KGM powder into water and stirring (600 rpm) at 25 °C for 2 h.

Referring to the method of Biller [7] with appropriate modification, aqueous phase ingredients (30% *w*/*w*) with varying concentrations of KGM or WP were prepared according to Table 1. Initially, aqueous phase ingredients were poured into TM5 Thermomix (Vorwerk Corporation, Wuppertal, Germany) and mixed uniformly at 1000 rpm. Then, the rotational speed was adjusted to 3000 rpm, and the oil phase was slowly dripped in. The rate of oil phase addition increased as the sample became sticky and milky. After stirring for 2 min, the samples hanging on the wall of machine were scraped off, and the fabrication was completed after continuing stirring for 1 min. The total oil phase was 70% *w*/*w* as a dispersed phase, and the total aqueous phase was 30% *w*/*w* as a continuous phase. The final concentration of WP and KGM in the total system were 0–3.0% *w*/*w* and 0–0.5% *w*/*w*, respectively. A comparative analysis was conducted between mayonnaise-like emulsions and commercial mayonnaise (70% *w*/*w* flaxseed oil and 10.0% *w*/*w* whole egg as emulsifier). 

### 2.3. Droplet Size Distribution and Mean Droplet Size Measurements

The droplet size distribution and mean droplet size measurements (D_4,3_;D_3,2_) of the samples were measured using Mastersizer 2000 (Malvern Instruments, Malvern, UK). The refractive indexes of flaxseed oil and water used in calculations were 1.474 and 1.33, respectively.

### 2.4. Microstructure Analysis

The microstructure of the samples was observed using CX40 metallographic microscope (Ningbo Shunning Instrument, Ningbo, China) equipped with a digital camera. A drop of the diluted emulsion sample was placed on a microscope slide and then covered with a coverslip. The micrographs of the samples were captured at 400× magnification.

### 2.5. Appearance Texture and Stackability

To observe the appearance texture and stackability of the emulsion, it was squeezed onto the slide using a dropper, and its appearance texture was recorded after 5 min. Additionally, the sample was drawn on the slide using a disposable plastic head dropper, and the letter “Z” was left for 5 min to observe its extrusion formation and stackability [5]. This method used to characterize the texture and extrusion characteristics of mayonnaise-like sauce was simple and intuitive, and it was also suitable for the actual process of extrusion from bottles when the system was applied to semi-solid condiments.

### 2.6. Rheological Properties

A Kinexus Lab+ rheometer (Malvern Instruments, Malvern, UK) equipped with a cone plate (40 mm, and 4°) was employed to characterize the rheological properties of the emulsion. The test was conducted at 25 °C. After loading the sample, two minutes were required for the sample to return to its original state. After the sample was loaded onto the stage, the viscosity of emulsion was recorded at the range of 0.1~100 s^−1^ shear rate. To assess the non-linear relationship between shear stress and shear rate of emulsions and obtain the rheological parameters, such as consistency coefficient (K) and flow index (n), the Herschel–Bulkley model (Y = Y_0_ + Kx^n^) (Y: shear stress; x:shear rate) was used. Before the dynamic frequency sweeping experiments, the linear viscoelastic regions (LVR) were defined using the strain sweep test: angular frequency, 1.0 rad/s, amplitude strain 0.01–10%. According to the LVR, the subsequent frequency sweeping was performed at 0.5% stain, and the elastic modulus G′ and viscous modulus G″ were recorded at the range of 0.628~62.8 rad/s. The correlation between G′ and G″ was determined using the power law model (G′ = G_0_′∙ω^n′^ and G″ = G_0_″∙ω^n^″), where G′and G″ were the storage modulus (Pa) and loss modulus (Pa), respectively; G_0_′ and G_0_″ were the storage modulus (Pa) and loss modulus (Pa) at angular frequency 1.0 rad/s, respectively; ω was the angular frequency (rad/s); n′ and n″ indicated the frequency dependence of the elastic modulus and loss modulus, respectively.

### 2.7. Statistical Analysis

All experiments were repeated three times. The results were expressed as means ± standard deviations (SD). Data and figures were processed using Excel and origin 2022. The differences between samples were calculated using LSD (SPSS 22.0). The letters a–d or A–D indicate significant differences between the samples (*p* < 0.05).

## 3. Results and Discussion

### 3.1. Stabilization of Emulsion

#### 3.1.1. Impact of KGM Concentration

##### Droplet Size

**Figure 1:**
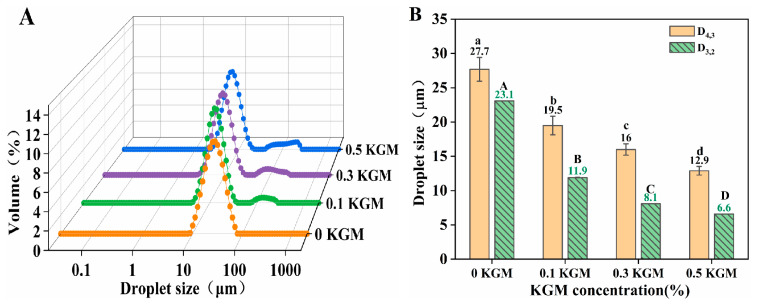
Effects of konjac glucomannan (KGM) concentration on (**A**) droplet size distribution and (**B**) volume-weighted average droplet size (D_4,3_) and surface area droplet size (D_3,2_) of mayonnaise-like emulsion. Different capital letters (a–d) and lowercase letters (A–D) indicate significantly different (LSD, *p* < 0.05) average droplet size (D_4,3_) and surface area droplet size (D_3,2_), respectively. Temperature = 25 °C.

Distribution and mean size of droplets were crucial parameters in assessing the physical stability of the emulsion system. The impact of KGM concentration (at WP concentration 2.0% *w*/*w*) on the distribution and mean droplets size of emulsions was characterized (Figure 1). For samples with no KGM (0 KGM), maximum D_4,3_ values of 27.7 μm and D_3,2_ values of 23.1 μm were obtained, and they showed uniform unimodal distributions. However, emulsions with varying concentrations of KGM had smaller D_4,3_ and D_3,2_ values but a bimodal distribution (a main peak located in small droplet size region and a shoulder peak located in a large droplet size region) (Figure 1A), indicating lower droplet uniformity.

Furthermore, with the increase in KGM concentration, D_4,3_ and D_3,2_ were significant decreased from 27.7 μm to 12.9 μm and from 23.1 μm to 6.6 μm, respectively. Although the minimum D_4,3_ of 12.9 μm and D_3,2_ of 6.6 μm were obtained at KGM 0.5% *w*/*w*, a wider shoulder peak located in the large droplet size region (>100 μm) was measured. This was because KGM was a non-adsorbed linear neutral polysaccharide, which increased the viscosity and steric hindrance effect of the system. The increase in viscosity had two effects on the droplet size of the emulsion: (1) Due to the high viscosity and large steric hindrance effect, the coalescence, aggregation, and agglomeration of oil droplets in the system were inhibited, leading to a reduction in droplet size and an improvement in the stability of the emulsion. (2) In the process of mayonnaise-like emulsion preparation, excessive viscosity was not conducive to the dispersion of the oil phase and the adsorption of emulsifiers at the oil–water interface, which increased droplet size, resulting in an uneven droplet size distribution. Therefore, the addition of KGM could reduce the droplet size of emulsion, but if the concentration is too high, the uniformity of droplet size will be reduced. In addition, the appearance of the shoulder peak located in the larger droplet size range might also be due to the uneven dispersion caused by the high viscosity of sample during droplet size test, as supported by the microstructure observations.

##### Visual Appearance, Texture, and Microstructure

**Figure 2:**
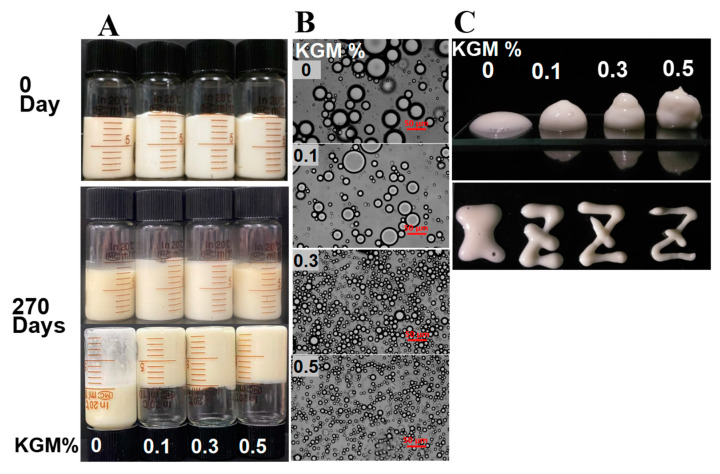
Effects of konjac glucomannan (KGM) concentration on appearance and texture of emulsion: (**A**) appearance; (**B**) microstructure; (**C**) stackability. Scale bar is 50 μm.

Stability of the mayonnaise-liked emulsion was observed visually by taking photos 0 days and 270 days after preparation. All the samples with different KGM concentrations were found to be stable (Figure 2A), even after 9 months of storage. Although there were no visible differences in the appearance of the samples, their textures and microstructures were noticeably different. If emulsions had stacking properties after extrusion, they were considered to possess a gel-like behavior [5]. As shown in Figure 2C, at 0–0.5% of KGM fractions, all samples displayed uniform texture. Notably, the emulsion stabilized by WP alone (in the absence of KGM) was flowable, while those co-stabilized by WPI and KGM exhibited a distinct self-supporting texture. Furthermore, with the KGM concentration increasing from 0.1% to 0.5%, the stackability and extrusion formability of samples were improved, indicating that the presence of KGM with increasing concentrations facilitated gel-like network formation [29].

The strengthening effect of the emulsions due to the presence of KGM could also be augmented by their microstructure (Figure 2B). With increasing KGM concentration, the droplet size of samples gradually decreased. This result was in good accordance with Figure 1. Moreover, droplets became more tightly packed as the KGM concentrations increased. It had been reported that an increase in packing density could increase the interactions of droplets and the viscosity of emulsion, promoting the formation of network structure [30].

#### 3.1.2. Impact of WP Concentration

##### Droplet Size

**Figure 3:**
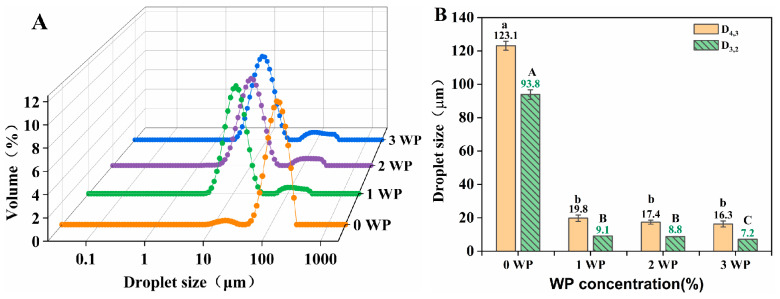
Effects of whey protein (WP) concentration on (**A**) droplet size distribution and (**B**) volume-weighted average droplet size (D_4,3_) and surface area droplet size (D_3,2_) of mayonnaise-like emulsion. Different capital letters (a,b) and lowercase letters (A–C) indicate significant differences (LSD, *p* < 0.05) in average droplet size (D_4,3_) and surface area droplet size (D_3,2_), respectively. Temperature = 25 °C.

Figure 3 shows droplet size distribution of emulsion with different WP concentration at KGM 0.3% *w*/*w*. In the absence of WP (0 WP), emulsion had a main peak located in the large droplet size range with a shoulder peak located in the small droplet size range. A maximum D_4,3_ 123.1 μm and a maximum D_3,2_ 93.8 μm were recorded, suggesting that the droplet size of the system was large and uneven. KGM, a hydrophilic polysaccharide, could not stabilize the oil-water interface alone. However, in the presence of WP, the main peaks of emulsions were all located in the smaller droplet size range, and the average droplet size of samples significantly decreased to less than 20 μm (D_4,3_) and 10.0 μm (D_3,2_).

However, the average droplet size of the samples with different WP concentrations showed no significant difference. When the emulsifier amount was sufficient to cover the oil droplets, the concentration of emulsifier in aqueous phase did not significantly affect droplet size [31]. Therefore, in this work, 1.0% WPI was found to be sufficient to cover the oil–water interface formed by a 70% oil phase.

**Figure 4:**
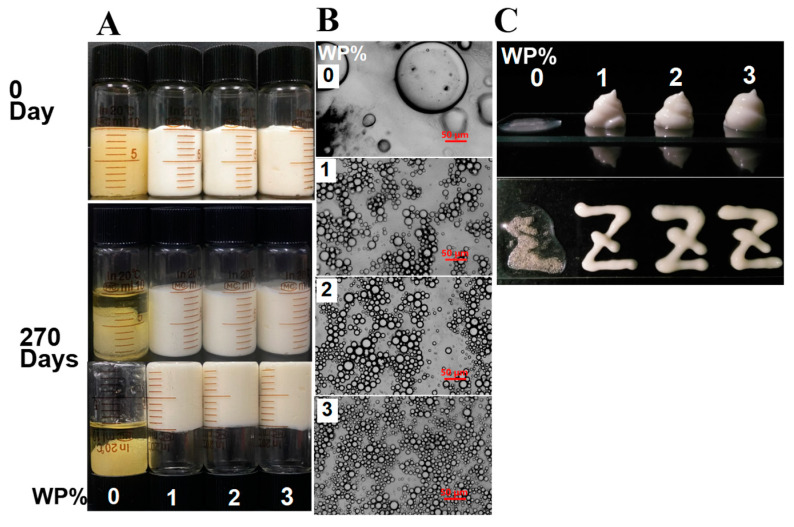
Effects of whey protein (WP) concentration on appearance and texture of emulsion: (**A**) appearance; (**B**) microstructure; (**C**) stackability. Scale bar is 50 μm.

The impact of WP concentration on the visual appearance, texture, and microstructure of the emulsion was observed (Figure 4). It was found that using KGM alone at c = 0.3% (in absence of WP) resulted in an unstable emulsion system, with free oil still noticeable and an oily yellow visual appearance (Figure 4A). This was consistent with the understanding that KGM was a water-soluble hydrophilic colloid with weak surface activity. Microstructure and stackability results further showed that in the absence of WP, the system was unstable, with large particle size. However, samples co-stabilized by WP and KGM at different WP concentrations displayed a uniform milky white color and showed excellent stacking and extrusion properties (Figure 4B,C), indicating their effectiveness in forming a gel-like emulsion.

##### Visual Appearance, Texture, and Microstructure

In fact, as previously mentioned in Section 3.1.1, the formation of small oil droplets in the continuous phase with high viscosity was a crucial factor in the preparation of a stable emulsion to prevent coalescence. The microstructure results indicated that emulsions stabilized by KGM alone contained large droplets, whereas emulsions co-stabilized by KGM and WP had noticeably smaller droplets. This was observed regardless of the WP concentration and was in good accordance with Figure 3.

### 3.2. Rheological Behavior of Mayonnaise-like Emulsion

In this study, steady flow and dynamic viscoelasticity were used to test the rheology of the emulsion. The Herschel–Bulkley model (Y = Y_0_ + Kx^n^) (Y: shear stress; x: shear rate) was widely used to assess the non-linear relationship between shear stress and shear rate of emulsions and to determine rheological parameters, such as consistency coefficient (K) and flow index (n). The rheological parameters of samples were depicted in Table 2 and Table 3. The determination coefficient (R^2^) was higher than 0.9, indicating that the Herschel–Bulkley model showed a good fit to the experimental data.

#### 3.2.1. Flow Behavior

##### Impact of KGM Concentration

**Figure 5:**
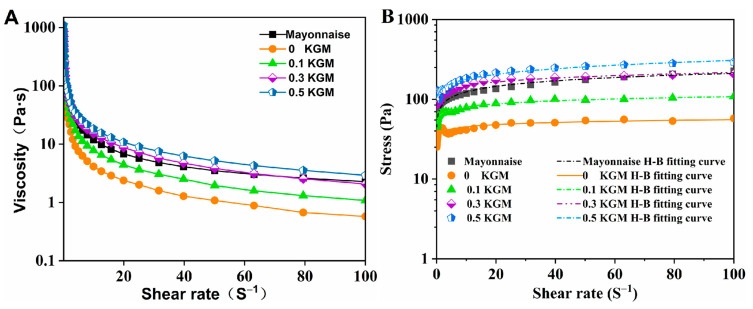
Rheological characteristics of mayonnaise-like emulsions prepared with different concentrations of konjac glucomannan (KGM): the apparent viscosity curves dependent on shear rate (**A**), the change of shear strain dependent on shear rate (**B**). Temperature = 25 °C.

The effect of different concentrations of KGM on the changes in apparent viscosity and shear stress of emulsions containing 2.0% *w*/*w* WP was shown in Figure 5.

The results revealed that apparent viscosity of all samples decreased with an increase in shear rate, indicating strong shear thinning behavior (Figure 5) and pseudoplastic fluids (n < 1) (Table 2), which is in keeping with previous studies on the rheology of mayonnaise-like sample [2,13]. The microstructure of systems was destroyed due to shear action, with the degree of damage increasing as the shear rate increased. At shear rate of 100 s^−1^, the shear viscosities of samples were all reduced to lower than 10 Pa∙s, indicating that the microstructure of the network was seriously damaged and that the new microstructure of the network cannot be formed in a short time [26]. Additionally, the viscosity and shear stress of emulsions increased with increasing KGM concentration in the range of 0.1–100 s^−1^ shear rate.

In addition, as the KGM concentration increased, the consistency coefficient (K) increased, while flow index (n) decreased. This could be attributed to several factors, including (1) KGM increasing the viscosity of the continuous phase; (2) as the oil droplets size decreased, the mean distances between them were smaller and the interactions between them were stronger, leading to a viscosity increase [32]; (3) the hydrogen bond interaction between KGM and WP might be another reason for the increased viscosity. The rheological properties of the emulsion at KGM 0.3% *w*/*w* were similar to the commercial mayonnaise.

Impact of WP Concentration

**Figure 6:**
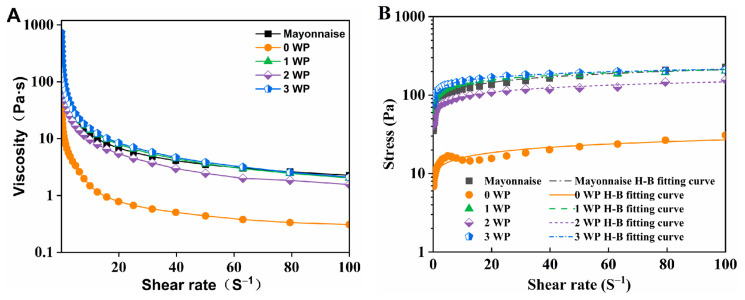
Rheological characteristics of mayonnaise-like emulsions prepared with different concentrations of whey protein (WP): the apparent viscosity curves dependent on shear rate (**A**), the change of shear strain dependent on shear rate (**B**). Temperature = 25 °C.

The apparent viscosity and shear stress of emulsions (KGM c = 0.3% *w*/*w*) with varying WP concentrations were plotted in Figure 6. The parameters from the Herschel–Bulkley fit were listed in Table 3.

The samples, including commercial mayonnaise, exhibited strong shear thinning behavior (Figure 6) and a pseudo-plastic behavior since the values of flow behavior index (n) were less than 1 (n < 1) (Table 3). In the range of 0.1–100 s^−1^ shear rate, when WP was absent (0 WP), the viscosity and shear stress were both minimum. These results were consistent with the findings in appearance and droplet size, indicating KGM could not stabilize the emulsion alone. Nevertheless, when WP was present, there was a sight dependence of viscosity and shear stress on WP concentration. With an increase in WP concentration from 0 to 3.0% *w*/*w*, Herschel–Bulkley fitting parameters of samples were generally increased (K, 5.29–95.87 Pa s^n^) and decreased (n, 0.31–0.15), respectively. The increase in K value and decrease in n value with increasing WP concentrations suggested that WP molecules bridged droplet to droplet, enhancing the strength of the interactions between droplets [6,33]. Although the increase was at minimum 2.0% *w*/*w*, the emulsion at this WP concentration showed a similar trend to commercial mayonnaise.

#### 3.2.2. Viscoelastic Properties

**Figure 7:**
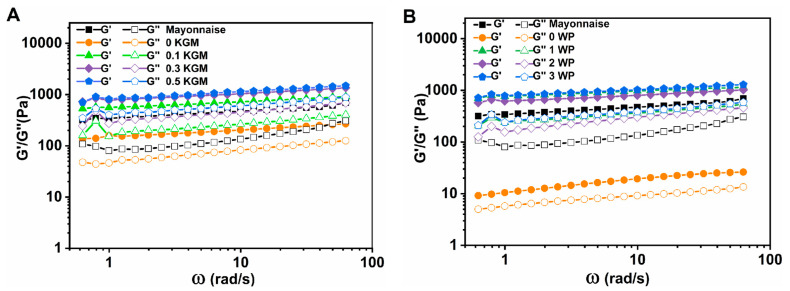
The change in elastic modulus (G′) and viscous modulus (G″) dependent on frequency (ω) of mayonnaise-like emulsion prepared with different concentrations of konjac glucomannan (KGM) (**A**) and whey protein (WP) (**B**). Temperature = 25 °C.

Figure 7 demonstrated the change in storage (G′) and loss (G″) modulus values with frequency for emulsions containing different KGM and WP concentrations. In general, when the G′ value is expected to be independent of frequency and G′ > G″, the emulsion is defined as a gel-like behavior [32]. As can be seen in Figure 7, in the range of 0.628–62.8 rad/s frequency, the storage modulus (G′) was consistently higher than the loss modulus (G″), which indicated that the emulsion samples presented a gel-like behavior. Moreover, G′ rose with the in KGM concentration (Figure 7A) and WP concentration (Figure 7B). This result confirmed the predominant role of KGM and WP concentration in the structure formation of gel-like emulsion, as we discussed earlier. It should be noted that for the sample without WP (0 WP), a stable emulsified system could not be formed, and its property of gel-like behavior was mainly due to KGM. A slight frequency dependence of G′ was observed, which is typical of protein-stabilized emulsions. This could be attributed to the development of elastic networks resulting from extensive bridging and flocculation processes [6,34].

The viscoelasticity of the mayonnaise-like emulsion was due to a network format, which was related to egg yolk proteins located between the interfaces of adjacent oil droplets [1] and also might be due to the hydrophobic interaction between lipids in egg yolk and oil droplets. In this work, the viscoelastic property (where G′ > G″) of the emulsions co-stabilized by KGM and WP was similar to that of commercial mayonnaise, suggesting that a KGM and WP mixture could effectively help compensate for the absence of egg yolk and stabilize the oil droplet interface. Moreover, elastic interfacial networks among oil droplets were found to be enhanced as the concentration of KGM and WP increased, as evidenced by the increase in G′ and G″ of all samples with increasing concentrations of KGM and WP.

In order to further ascertain the time-stability of gel and gel-like network, the power law model was used to fit G′ and G″ with ω to obtain the related rheological parameters [35,36,37,38]. The results indicated that the emulsion samples had gel-like characteristics due to their G′ and G″ being able to be fitted to the angular frequency (ω) using the power law model (R^2^ > 0.9) (Table 4). Except for 0 KGM and 0 WP, both G_0_′ and G_0_″ parameters (G_0_′ and G_0_″ are the storage and loss moduli at 1 rad/s, respectively) were noticeably greater in emulsion samples vs. the commercial mayonnaise, indicating greater gel strength [36]. The n′ value of all emulsion samples was lower than 1, also indicating a gel-like property [37]. Additionally, although there was no significant difference, n′ value generally decreased from 0.14 to 0.10 and from 0.22 to 0.10 as the concentrations of KGM and WP increased, respectively, indicating that the emulsion sample gradually developed a semi-solid form [38]. These results were consistent with physical stability and static viscosity results. In addition, the n′ value of all emulsion samples was less than the n″ value, meaning the rate of decrease in G′ was lower than in G″ and that the gel-like network was slightly reinforced with decreasing angular frequency [35].

Taken together, emulsions stabilized with a KGM-WP mixture had similar rheological behaviors to commercial mayonnaise, indicating that mayonnaise-like emulsion with high viscoelasticity, consistency, and texture could be fabricated by replacing egg yolk with a KGM-WP mixture.

## 4. Conclusions

This study proposed a strategy to prepare mayonnaise-like oil-in-water emulsions that can potentially replace traditional mayonnaise. The strategy involved the usage of mixtures solution of whey protein (WP) and konjac glucomannan (KGM) as the continuous phase and flaxseed oil as dispersed phase to fabricate emulsion. The impact of different concentrations of WP and KGM on droplet size, texture, microstructure and rheological properties of emulsion were characterized. The results showed that increasing the the concentrations of KGM (0.1–0.5% *w*/*w*) and WP (1.0–3.0% *w*/*w*) progressively decreased the oil droplet size. The static flow behavior revealed that emulsions showed a phenomenon of pseudo-plastic fluid characteristics. The dynamic rheological properties indicated that G′ was always higher than G″ in the range of 0.628–62.8 rad/s angular frequency. Moreover, apparent viscosity, G′, and G″ showed increasing trends with increases in KGM and WP concentrations, thereby leading to an enhancement in the network structure. The network of emulsion co-stabilized by KGM and WP might be maintained through three aspects: (1) an increase in continuous phase viscosity, primarily from the contribution of KGM; (2) the excellent emulsifying activity of WP molecules; (3) the noncovalent interactions between KGM and WP. Furthermore, emulsions stabilized by a KGM-WP mixture exhibited shear thinning behavior and viscoelastic properties, which was similar to the commercial mayonnaise. These results suggested that the KGM-WP mixtures could effectively replace egg yolk to produce cholesterol-free mayonnaise-like emulsions. These results had significant implications for the development of protein–polysaccharide mixtures as promising egg yolk replacers in modification of traditional mayonnaise. However, thixotropy behavior and the oxidation stability of mayonnaise-like high-viscosity emulsions rich in unsaturated fatty acids (flaxseed oil), which are very important in practical applications, need to be further studied for future applications.

## Figures and Tables

**Table 1:** The aqueous phase composition (% *w*/*w*) of the emulsion. Total aqueous phase was 30% *w*/*w*. Konjac glucomannan, KGM; whey protein, WP.

Samples		Aqueous Phase Ingredients (%)			
		30% WP Stock Solution	5% KGM Stock Solution	Double-Distilled Water	Condiments
WP%	0 WP	0	6.0	16.0	The proportion of total condiments was 8.0%: 4.0% vinegar; 2.0% sugar; 2.0% salt
	1 WP	3.3	6.0	12.7	
	2 WP	6.7	6.0	9.3	
	3 WP	10.0	6.0	6.0	
KGM%	0 KGM	6.7	0	15.3	
	0.1 KGM	6.7	2.0	13.3	
	0.3 KGM	6.7	6.0	9.3	
	0.5 KGM	6.7	10.0	5.3	

**Table 2:** Herschel–Bulkley equation fitting parameters of shear strain curve with shear rate for samples with different konjac glucomannan (KGM) concentrations.

Samples	K/Pa s^n^	n	R^2^
0 KGM	36.70 ± 6.34 c	0.49 ± 0.08 a	0.90
0.1 KGM	37.79 ± 5.48 c	0.40 ± 0.03 a	0.98
0.3 KGM	68.35 ± 3.68 b	0.24 ± 0.04 b	0.97
0.5 KGM	84.65 ± 3.69 a	0.17 ± 0.05 c	0.96
Mayonnaise	65.69 ± 5.59 b	0.25 ± 0.04 b	0.98

Different letters (a–c) in the same column mean significantly differences (LSD, *p* < 0.05) fitting parameters between different konjac glucomannan (KGM) concentrations.

**Table 3:** Herschel–Bulkley equation fitting parameters of shear strain curve with shear rate for samples with different whey protein (WP) concentrations.

Sample	K/Pa s^n^	n	R^2^
0 WP	5.29 ± 2.06 d	0.31 ± 0.07 a	0.84
1 WP	89.14 ± 8.21 b	0.18 ± 0.01 c	0.99
2 WP	61.51 ± 7.32 c	0.24 ± 0.02 b	0.99
3 WP	95.87 ± 7.85 a	0.15 ± 0.01 d	0.99
Mayonnaise	63.23 ± 6.57 c	0.25 ± 0.03 b	0.98

Different letters (a–d) in the same column indicate significant differences (LSD, *p* < 0.05) between samples different with whey protein (WP) concentrations.

**Table 4:** Fitting parameters to the power law equation for all the emulsions with different konjac glucomannan (KGM) concentrations and whey protein (WP) concentrations.

Sample	G_0_′ (Pa)	n′	R^2^	G_0_″ (Pa)	n″	R^2^
0 KGM	147.4 ± 0.9 e	0.14 ± 0.01 ab	0.99	34.1 ± 0.3 e	0.21 ± 0.01 b	0.99
0.1 KGM	555.6 ± 2.8 c	0.12 ± 0.01 bc	0.99	114.2 ± 5.8 c	0.16 ± 0.02 c	0.90
0.3 KGM	681.0 ± 7.6 b	0.11 ± 0.01 bc	0.98	183.5 ± 8.0 b	0.17 ± 0.02 c	0.91
0.5 KGM	815.9 ± 10 a	0.10 ± 0.01 c	0.97	238.7 ± 6.2 a	0.16 ± 0.01 c	0.94
Mayonnaise	336.1 ± 5.5 d	0.16 ± 0.01 a	0.97	48.4 ± 2.7.2 d	0.29 ± 0.02 a	0.91
0 WP	11.8 ± 0.2 E	0.22 ± 0.01 A	0.98	4.9 ± 0.04 D	0.18 ± 0.01 C	0.99
1 WP	769.6 ± 5.4 B	0.12 ± 0.01 C	0.99	147.9 ± 2.8 B	0.17 ± 0.01 C	0.97
2 WP	615.9 ± 5.8 C	0.11 ± 0.02 C	0.99	106.4 ± 2.1 A	0.18 ± 0.01 C	0.98
3 WP	787.1 ± 6.3 A	0.10 ± 0.01 C	0.99	154.1 ± 4.2 B	0.17 ± 0.01 C	0.95
Mayonnaise	336.1 ± 5.5 D	0.16 ± 0.01 B	0.97	48.4 ± 2.7.2 C	0.29 ± 0.02 A	0.91

Different lowercase letters (a–e) and capital letters (A–E) in the same column indicate significant differences (LSD, *p* < 0.05) between samples with different konjac glucomannan (KGM) concentrations and whey protein (WP) concentrations, respectively.

## Data Availability

The data presented in this study are available on request from the corresponding author.
